# *Toxoplasma* metabolic flexibility in different growth conditions

**DOI:** 10.1016/j.pt.2022.06.001

**Published:** 2022-09

**Authors:** Daniel Walsh, Nicholas J. Katris, Lilach Sheiner, Cyrille Y. Botté

**Affiliations:** 1 Wellcome Centre for Integrative Parasitology, University of Glasgow, Glasgow, UK; 2 ApicoLipid Team, Institute for Advanced Biosciences, CNRS UMR5309, Université Grenoble Alpes, INSERM U1209, Grenoble, France

**Keywords:** Apicomplexa, *Toxoplasma gondii*, metabolism, metabolic adaptation, lipids, host–parasite metabolic interactions

## Abstract

Apicomplexan parasites have complex metabolic networks that coordinate acquisition of metabolites by *de novo* synthesis and by scavenging from the host. *Toxoplasma gondii* has a wide host range and may rely on the flexibility of this metabolic network. Currently, the literature categorizes genes as essential or dispensable according to their dispensability for parasite survival under nutrient-replete *in vitro* conditions. However, recent studies revealed correlations between medium composition and gene essentiality. Therefore, nutrient availability in the host environment likely determines the requirement of metabolic pathways, which may redefine priorities for drug target identification in a clinical setting. Here we review the recent work characterizing some of the major *Toxoplasma* metabolic pathways and their functional adaptation to host nutrient content.

## Highlights

Apicoplast fatty-acid synthase complex (FASII) genes in apicomplexan parasites are essential but only under low host lipid content.
*Toxoplasma* infection is affected by the availability of host nutrients, which determines the essentiality of parasite metabolic pathways.
Some parasite metabolic genes are essential only under starvation, which redefines the requirements of essentiality of a metabolic pathway as specific to the physiological states and fluctuations of the host environment.

## The versatility of the *Toxoplasma* parasite

*Toxoplasma gondii* is a member of the apicomplexan phylum of protozoan parasites and is the causative agent of toxoplasmosis. This phylum also includes the genus *Plasmodium*, species of which are the causal agents of malaria, a disease that is endemic in many tropical and subtropical countries and which is responsible for the deaths of approximately 627 000 people each year, mainly children under the age of five [1]. *T. gondii* is a lethal threat for the fetus of women infected for the first time during pregnancy as well as for immunodeficient patients, such as those with HIV-AIDS, patients undergoing chemotherapy, and patients taking immunosuppressive drugs for organ transplants [2,3]. However, this zoonotic pathogen typically presents as an asymptomatic chronic infection in approximately one-third of the world’s population and is thus recognized as a neglected disease [3]. Humans are intermediate hosts for *T. gondii* and so only the asexual life cycle takes place in which two life stages of the parasite may persist and interconvert [4]; these two stages are the tachyzoite, the rapidly dividing life stage responsible for the acute phase of the disease, and the slower growing, but immune-evasive, chronic bradyzoite [4].

The *T. gondii* life cycle includes growth in two types of host: a definitive feline host, in which sexual division occurs, and an intermediate host (warm-blooded animals, including humans) in which the parasite divides asexually [4]. Several birds, as well as land and aquatic mammals, have also been identified as *T. gondii* reservoirs [5]. This very wide intermediate host range and multiplicity of tissues infected, expose *T. gondii* to diverse host environments, indicating that the parasite needs to be metabolically flexible in order to adapt to these changes and to properly propagate and survive. It is foreseeable that *T. gondii* might not require all parts of a metabolic pathway if a nutrient is abundant in the host cell. Therefore, an expanded metabolic network is evolutionarily conserved likely due to selective pressure of diverse host availability even where a pathway is not essential for every type of intermediate host (or types of host cells within a single animal host). Hence, some metabolic pathways or their components are essential or dispensable under certain nutritional and environmental conditions and can be activated, inhibited, or reprogrammed upon the parasite sensing conditional changes. This expands on recent evidence that metabolic pathways are in fact essential only when parasites are grown in low-nutrient *in vitro* conditions [6., 7., 8., 9.]. Here we review some of the major metabolic pathways on which *T. gondii* depends for survival, and we interrogate the metabolic flexibility in the context of nutrient availability to explore the physiological relevance of *in vitro* systems during infection.

## The role of pyruvate kinase in parasite metabolism

**Figure 1:**
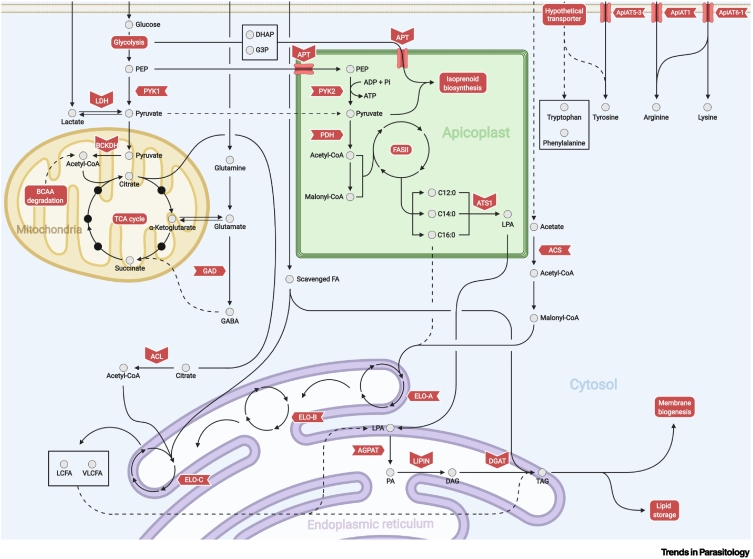
A schematic overview of various *Toxoplasma gondii* metabolic pathways. This diagram highlights several substrate-sharing metabolic pathways, including: glycolysis, anaerobic glycolysis, the TCA cycle, glutaminolysis, the GABA shunt, FASII, FA elongation, bulk phospholipid synthesis, novel amino acid transporters, and associated metabolite scavenging. Abbreviations: acetyl-CoA, acetyl-coenzyme A; ACL, ATP-citrate lyase; ACS, acetyl-coenzyme A synthetase; ADP, adenosine diphosphate; AGPAT, acyl-glycerol 3-phosphate acyltransferase; ApiAT, apicomplexan amino acid transporter; APT, apicoplast phosphate transporter; ATP, adenosine triphosphate; ATS, apicoplast glycerol 3-phosphate acyltransferase; BCAA, branched-chain amino acid; BCKDH, branched-chain ketoacid dehydrogenase; C12:0, laurate; C14:0, myristate; C16:0, palmitate; DAG, diacylglycerol; DGAT, diacylglycerol-acyltransferase; DHAP, dihydroxyacetone phosphate; ELO, elongase; FA, fatty acid; G3P, glyceraldehyde 3-phosphate; GABA, γ-aminobutyric acid; GAD, glutamate decarboxylase; FASII, fatty-acid synthesis II pathway; LCFA, long-unsaturated-chain fatty acids; LDH, lactate dehydrogenase; LPA, lysophosphatidic acid; PA, phosphatidic acid; PDH, pyruvate dehydrogenase; PEP, phosphoenolpyruvate; Pi, inorganic phosphate; PYK, pyruvate kinase; TAG, triacylglycerol; TCA, tricarboxylic acid; VLCFA, very-long-unsaturated-chain fatty acids. Figure created with BioRender.com.

Pyruvate is a key intermediate and precursor in central carbon metabolism and an important metabolic intersection for many different pathways, including the mitochondrial tricarboxylic acid (TCA) cycle, the apicoplast fatty-acid synthase complex (FASII), isoprenoid precursor synthesis pathway (also known as apicoplast DOXP pathway), and cytosolic anaerobic glycolysis (Figure 1) [10,11]. As a primary source of energy, glucose scavenged from the host is converted to pyruvate via the parasite’s glycolytic pathway (Figure 1) [12]. However, when available in the extracellular culture environment, other metabolites, such as glutamine and lactate, can be incorporated into pyruvate via glutaminolysis and anaerobic glycolysis respectively (Figure 1) [12]. In the steps of the glycolytic pathway, pyruvate kinase (PYK) converts phosphoenolpyruvate (PEP) into pyruvate (Figure 1) [11]. The *T. gondii* genome encodes two variants of PYK. A cytosolic *Tg*PYK1 takes on its canonical role in fueling the mitochondrial TCA cycle, whilst an apicoplast-located *Tg*PYK2 potentially uses pyruvate to fuel both the isoprenoid precursor pathway (i.e., isopentenylpyrophosphate IPP, DMAPP) and the FASII pathway in the apicoplast, both critical for parasite survival (Figure 1) [10]. For this reason, *Tg*PYK2 was expected to be essential for parasite survival [10]. However, a *Tg*PYK2 **knockout** (**KO**) (see Glossary) mutant (Δ*Tg*PYK2) grown in high-nutrient conditions (high glucose, high nutrient, and lipids) (Table 1) [10] showed no change in growth rate or viability in cell culture, nor *in vivo* where 100% mortality in infected immunocompetent mice occurred [10].

**Table 1:** Essentiality of *Toxoplasma gondii* metabolic mutants based on nutrient availability and media content^a^

Inhibited gene of interest	Methodology described in	Culturing conditions	Derived parasite strain	Host cell line	Culture media	Serum type and concentration	Incubation temperature	Incubation CO_2_ concentration	Antibiotics and concentration	Supplement and concentration	Essentiality
*Tg*PYK1 and *Tg*PYK2	Xia *et al.* (2019) [10] Shen *et al.* (2017) [82] Roos *et al.* (1995) [83]	Control	RH	HFF	MEM	1% heat-inactivated FBS	37°C	5%	–	–	PYK2 becomes essential where PYK1 is inhibited
*Tg*PYK1 and *Tg*PYK2	Xia *et al.* (2019) [10] Shen *et al.* (2017) [82] Roos *et al.* (1995) [83]	High-nutrient conditions	RH	HFF	MEM	1% heat-inactivated FBS	37°C	5%	–	8 mM lactate + 8 mM glutamine, or 4500 mg/l glucose + 8 mM alanine	Not essential
*Tg*BCKDH-E1a	Oppenheim *et al.* (2014) [19]	Propagation	RH	HFF	DMEM	5% FCS	37°C	5%	25 μg/l gentamicin	2 mM glutamine	–
*Tg*BCKDH-E1a	Oppenheim *et al.* (2014) [19]	Control	RH	HFF	DMEM 41966	5% FCS	37°C	5%	25 μg/ml gentamicin	6 mM glutamine	Not essential. Reduced growth rate and smaller plaques observed
*Tg*BCKDH-E1a	Oppenheim *et al.* (2014) [19]	Glucose depleted	RH	HFF	DMEM 11960	5% FCS	37°C	5%	25 μg/ml gentamicin	6 mM glutamine	Not essential. Severely reduced growth rate
*Tg*BCKDH-E1a	Oppenheim *et al.* (2014) [19]	Glutamine depleted	RH	HFF	DMEM 11966	5% FCS	37°C	5%	25 μg/ml gentamicin	–	Not essential. Reduced growth rate
*Tg*BCKDH-E1a	Oppenheim *et al.* (2014) [19]	Glucose depleted and high acetate	RH	HFF	DMEM 11960	5% FCS	37°C	5%	25 μg/l gentamicin	6 mM glutamine and 5 mM acetate	Not essential. Partially restored growth rate
*Tg*GAD	MacRae *et al.* (2012) [20]	Control	RH	HFF or hTERT-BJ1	DMEM	5% FBS	37°C	5%	–	–	Not essential
*Tg*GAD	MacRae *et al.* (2012) [20]	Control	PRU	HFF or hTERT-BJ1	DMEM	10% FBS	37°C	5%	–	–	Not essential
*Tg*ELO-A, B, or C	Ramakrishnan *et al.* (2012) [28] Striepen and Soldati (2007) [84]	Control	RH	HFF	DMEM	1% FCS	37°C	5%	1:200 of 10 000 units/l penicillin/ streptomycin	1:100 of 200 mM stock glutamine	Not essential. Effect of variable FBS not determined
*Tg*DEH	Ramakrishnan *et al.* (2015) [32] Striepen and Soldati (2007) [84]	Control	RH	HFF	DMEM	1% FCS	37°C	5%	1:200 of 10 000 units/l penicillin/ streptomycin	1:100 of 200 mM stock glutamine	Essential. Complete growth arrest after 5 days. Effect of variable FBS not determined
*Tg*DEH	Ramakrishnan, *et al.* (2015) [32] Striepen and Soldati (2007) [84]	High-nutrient conditions	RH	HFF	DMEM	1% FCS	37°C	5%	1:200 of 10 000 units/l penicillin/ streptomycin	1:100 of 200 mM stock glutamine and 250 μM mixture of [C18:1, C20:1, C22:1, and C24:1], or 250 μM of C20:1, or 250 μM of C22:1	Not essential when supplemented with unsaturated fatty acid mix
*Tg*LIPIN	Dass *et al.* (2021) [38]	Control	RH	HFF	DMEM	0%/1%/10% FBS (escalating lipotoxicity)	37°C	5%	25 μg/ml gentamicin	2 mM glutamine	Essential in 10% FBS, but more tachyzoite growth in reduced FBS
*Tg*NPT1 (*Tg*ApiAT1)	Rajendran *et al.* (2017) [8]	Control	RH	HFF	RPMI 1640	1% FBS	37°C	5%	Unspecified antibiotics	–	Essential in DMEM but not RPMI due to lack of amino acid availability
*Tg*NPT1 (*Tg*ApiAT1)	Parker *et al.* (2019) [68]	Nutrient deprived	RH	HFF	MAAM	1% FBS	37°C	5%	Unspecified antibiotics	–	Not essential. Reduced growth rate
*Tg*ApiAT5-3	Parker *et al.* (2019) [68]	Control	RH	HFF	DMEM	1% FCS	37°C	5%	Unspecified antibiotics	–	Not essential. Reduced growth rate and smaller plaques observed
*Tg*ApiAT5-3	Parker *et al.* (2019) [68]	Nutrient deprived	RH	HFF	MAAM	1% FCS	37°C	5%	Unspecified antibiotics	–	Essential
*Tg*ApiAT5-3	Parker *et al.* (2019) [68]	High-nutrient conditions	RH	HFF	MAAM	1% FCS	37°C	5%	Unspecified antibiotics	2.5 mM tyrosine	Not essential
*Tg*ApiAT6-1	Fairweather *et al.* (2021) [9]	Control	RH	HFF	RPMI 1640	1% FCS	37°C	5%	50 U/ml penicillin, 50 μg/ml streptomycin, 10 μg/ml gentamicin, and 0.25 μg/ml amphotericin B	2 mM glutamine	Essential. No rescue possible with arginine or lysine supplementation
*Tg*FabD	Liang *et al.* (2020) [43] Shen *et al.* (2017) [82] Roos *et al.* (1995) [83]	Control	RH	HFF	MEM	1% heat-inactivated FBS	37°C	5%	–	–	Not essential. Reduced growth rate and smaller plaques observed
*Tg*FabD	Liang *et al.* (2020) [43]	Isotope labelling	RH	HFF	Glucose free DMEM	1% heat-inactivated FBS	37°C	5%	–	8 mM glucose	Not essential in 10% FBS. Growth in variable FBS not determined
*Tg*ATS1	Amiar *et al.* (2020) [6]	High glucose	RH	HFF	DMEM 41965-062	1% FBS/10% FBS (complement)	37°C	5%	–	1 mM glutamine	Increased viability with high FBS
*Tg*ATS2	Amiar *et al.* (2020) [6]	High glucose	RH	HFF	DMEM 41965-062	1% FBS/10% FBS (complement)	37°C	5%	–	1 mM glutamine	Slightly reduced growth in low FBS
*Tg*ACP	Ramakrishnan *et al.* (2012) [28]	Control	RH	HFF	DMEM	1% FCS	37°C	5%	–	–	Essential. Growth in variable FBS not determined
*Tg*ASPV	Amiar *et al.* (2020) [6]	High glucose	RH	HFF	DMEM 41965-062	1%/10% FBS (complement)	37°C	5%	–	1 mM glutamine	Reduced plaques in 0% FBS, growth defect is exacerbated in reduced FBS
*Tg*MYR1	Amiar *et al.* (2020) [6]	High glucose	RH	HFF	DMEM 41965-062	1%/10% FBS (complement)	37°C	5%	–	1 mM glutamine	Reduced growth but consistent between 0, 1, and 10% FBS. Likely lack of host rewiring leads to less nutritious host cell
*Tg*ACBP1 and *Tg*SCP2	Amiar *et al.* (2020) [6]	High glucose	RH	HFF	DMEM 41965-062	1%/10% FBS (complement)	37°C	5%	–	1 mM glutamine	ACBP1 and SCP2 not essential in any FBS condition. Normal growth
*Tg*ACBP1 and *Tg*SCP2	Fu *et al.* (2019) [85]	Control	RH	HFF	DMEM	1% FBS	37°C	5%	10 units/ml penicillin, and 100 mg/ml streptomycin	–	ACBP1 and SCP2 not essential in 1% FBS. Moderately reduced growth rate
*Tg*ACBP2	Amiar *et al.* (2020) [6]	High glucose	RH and PRU	HFF	DMEM 41965-062	1%/10% FBS (complement)	37°C	5%	–	1 mM glutamine	Not essential in 0, 1, or 10% FBS
*Tg*ACBP2	Fu *et al.* (2019) [85]	Control	RH and PRU	HFF	DMEM	1% FBS	37°C	5%	10 units/ml penicillin, and 100 mg/ml streptomycin	–	Not essential in 1% FBS in RH Type I tachyzoites. Important for growth in Type II. Difference is apparently strain specific
*Tg*ACS	Amiar *et al.* (2020) [6]	High glucose	RH	HFF	DMEM 41965-062	1%/10% FBS (complement)	37°C	5%	–	1 mM glutamine	Not essential in 0, 1, or 10% FBS
*Tg*ACL and *Tg*ACS	Kloehn *et al.* (2020) [16]	Control	RH	HFF	DMEM	5% FCS	37°C	5%	25 μg/ml gentamicin	2 mM glutamine	Individually dispensable, essential together. Effect of high-nutrient conditions not determined

a Abbreviations: ACBP, acyl-coenzyme A binding protein; ACL, ATP citrate lyase; ACP, acyl carrier protein; ACS, acetyl-coenzyme A synthetase; ApiAT, apicomplexan amino acid transporter; ASP, aspartyl protease; ATS, apicoplast glycerol-3phosphate acyltransferase; BCKDH-E1a, branched-chain ketoacid dehydrogenase E1a subunit; DEH, hydroxyacyl-coenzyme A dehydratase; ELO, elongase; DMEM, Dulbecco’s Modified Eagle’s Medium; FBS, fetal bovine serum; FCS, fetal calf serum; GAD, glutamate decarboxylase; HFF, human foreskin fibroblast; hTERT-BJ1, human telomerase reverse transcriptase-immortalized BJ fibroblasts; MAAM, minimal amino acid medium; MEM, modified Eagles medium; MYR, Myc regulation; NPT, novel putative transporter; PYK, pyruvate kinase; RPMI, Roswell Park Memorial Institute; SCP, sterol carrier protein.

By contrast, a tetracycline inducible **knockdown** of *Tg*PYK1 (**i∆*Tg*PYK1**) resulted in a strong growth defect in similar high-nutrient conditions [10]. *Tg*PYK2 was then deleted to create an iΔ*Tg*PYK1**ΔPYK2** *T. gondii* double PYK mutant, which was fully lethal [10]. Ablation of *Tg*PYK1 also resulted in a slow loss of the apicoplast whilst the double *Tg*PYK mutant effectively lost the apicoplast [10]. Interestingly, the disruption of *Tg*PYK1 significantly reduced the production of fatty acids (FAs) generated via the apicoplast FASII where it was expected that *Tg*PYK2 was the main provider of carbons for fatty acid *de novo* synthesis [10]. Hence, *Tg*PYK1 and *Tg*PYK2 likely have complementary functions to maintain an active FASII pathway, with *Tg*PYK1 being the main provider and *Tg*PYK2 potentially being an accessory protein. Furthermore, this work strongly suggests that pyruvate can directly reach the apicoplast using an alternative route to the apicoplast phosphate transporter (APT) pathway. Indeed, previous biochemical characterization showed that the *Plasmodium falciparum* apicoplast triose phosphate transporter *Pf*TPT (APT in *T. gondii*) was only able to import PEP and triose phosphates (dihydroxyacetone phosphate, DHAP, and glyceraldehyde 3-phosphate, G3P), but not pyruvate, from the parasite glycolytic pathway so to provide the carbons for pyruvate generation and utilization by and within the apicoplast (Figure 1) [13].

Interestingly, the growth of the *Tg*PYK mutants could be partly rescued through supplementation of various metabolic substrates, such as lactate (Table 1). Both repressed iΔ*Tg*PYK1 and iΔ*Tg*PYK1ΔPYK2 mutants grew significantly faster when cultured with either 8 mM lactate or 8 mM alanine [10]; while addition of 8 mM pyruvate did not demonstrate a similar growth rate recovery, perhaps caused by less efficient import [10]. These observations suggested that lactate and alanine can be used as alternative carbon sources when PEP cannot be converted to pyruvate [10]. To test this hypothesis with regard to lactate, the authors explored the lactate dehydrogenase expressed in the tachyzoite stage. Lactate dehydrogenase 1 (LDH1), which is predominantly associated with anaerobic glycolysis [12], converts pyruvate into lactate (Figure 1) while recycling a single molecule of nicotinamide adenine dinucleotide (NAD^+^) in the process [10]. Deletion of *Tg*LDH1 in an iΔ*Tg*PYK1 mutant background, grown in lactate-supplemented media, resulted in negligible parasite growth when *Tg*PYK1 is repressed [10]. As for the role of alanine as an alternative source of pyruvate, *T. gondii* encodes an aminotransferase which may (per **LAMP** database [14]) catalyze the interconversion of L-alanine isomers and oxoglutarate to pyruvate and L-glutamate isomers. However, this has not been experimentally tested. Collectively, these data show how interwoven metabolic pathways can cooperate to produce the central metabolite pyruvate from a variety of sources, enabling metabolic adaptation in diverse host glucose environments, such as in muscle tissue where lactate build-up is likely more abundant than free glucose [15].

## Acetyl-coenzyme A (acetyl-CoA) and TCA cycle homeostasis

Apicomplexan parasites can synthesize acetyl-CoA, a central metabolite that is necessary to shuttle carbon around organellar compartments for various metabolic processes [16]. *T. gondii* is highly flexible in acetyl-CoA production and can make this central metabolite from various sources. Acetyl-CoA is unreasonably large to be transported by itself, so it is synthesized in various organelles, and these represent acetyl-CoA pools which facilitate the transfer of carbon as acetate (Figure 1). Acetyl-CoA produced by the parasite has multiple uses, including as a substrate for the mitochondrial TCA cycle, the endoplasmic reticulum (ER)-based fatty-acid-elongation pathway, apicoplast and cytoplasmic fatty-acid synthesis, and histone acetylation [16,17]. The *T. gondii* genome encodes one putative cytosolic acetyl-CoA synthetase (*Tg*ACS) which generates acetyl-CoA for both fatty-acid elongation at the ER and histone acetylation in the nucleus (Figure 1) [17]. Citrate generated in the TCA cycle can also be exported from the mitochondria and converted to acetyl-CoA by ATP citrate lyase (*Tg*ACL) in the cytosol (Figure 1) [16,18]. An apicoplast-resident pyruvate dehydrogenase (PDH) can produce acetyl-CoA using the organelle’s pyruvate pool (Figure 1) [17,19].

Finally, branched-chain ketoacid dehydrogenase (BCKDH) complex converts glycolytically derived pyruvate into acetyl-CoA in the mitochondrion (Figure 1) [19]. In other eukaryotes this reaction is catalyzed by mitochondrial PDH, but this enzyme isoform is absent in *T. gondii* [19,20]. Ablation of the *Tg*BCKDH-E1a subunit in *T. gondii* significantly reduced parasite replication rates *in vitro* in nutrient-replete media over 24 h, while maintaining full invasion and egress functionality (Table 1) [19]*.* When grown in glucose-depleted cell culture medium, the Δ*Tg*BCKDH tachyzoite growth could be partially restored with 5 mM acetate but not glutamine, consistent with a role for BCKDH in acetyl-CoA synthesis [19].

Despite having multiple sources of acetyl-CoA, the loss of *Tg*BCKDH severely reduces the overall levels of acetyl-CoA in *T. gondii* [19]. Characterization of a *Tg*BCKDH-E1a-deficient line identified a reduction in acetyl-CoA availability which coincided with a drop in protein acetylation, likely affecting downstream transcription of other protein-coding genes [16]. Indeed, evidence suggests that acetylation disruption, coupled with an overabundance of pyruvate, also triggers increased gluconeogenic activity, siphoning off remaining valuable energy [16].

The second most abundant source of carbon for *T. gondii* is typically glutamine [10]. Through glutaminolysis, glutamine is degraded to glutamate and can enter the mitochondrial TCA cycle either through interconversion to α-ketoglutarate via aspartate aminotransferases, or by glutamate dehydrogenase (Figure 1) [21,22]. In a study by MacRae *et al.*, the authors found another divergent TCA cycle feature in the Apicomplexa in which glutamine can enter the TCA cycle through another entry point: the γ-aminobutyric acid (GABA) shunt [20]. The enzyme glutamate decarboxylase (*Tg*GAD) catalyzes glutamate into GABA, and then via an uncharacterized mechanism, GABA can be converted to succinate (Figure 1) [20]. Similarly, a partial GABA shunt is present in *Plasmodium* and is nonessential in asexual stages [23]. In plants, through the sequential action of a GABA transaminase and succinate semialdehyde dehydrogenase (SSD), GABA is converted to intermediate SSD, and then to succinate [24]. SSD dehydrogenase is not predicted to exist in *Plasmodium* but is in *T. gondii* [23,25]. Although a GABA transaminase has not been described in *T. gondii*, ornithine transaminase (*Tg*OAT) has been suggested to mediate this role [26].

Genetic ablation of *Tg*GAD resulted in minimal change to *in vitro* parasite growth rates in nutrient-replete conditions with 10% **fetal bovine serum** (**FBS**) (Table 1) [20]. Egressed tachyzoite motility, however, was quickly inhibited when the mutants were suspended in carbon-deficient **phosphate-buffered saline** (**PBS**), unless supplemented with exogenous glutamine [20]. By comparison, wild-type *T. gondii* can glide under these conditions, likely using excess GABA as an energy reserve [20]. *In vivo*, mice mortality rates after infection with either RHΔ*Tg*GAD or type II cyst-forming PRUΔ*Tg*GAD parasites remained high [20]. By contrast to *Tg*GAD, the importance of *Tg*BCKDH in cyst-forming strains of *T. gondii* has not been explored. However, there is new evidence suggesting that the TCA cycle itself may be dispensable for the chronic life cycle stage [27].

Both *Tg*BCKDH and *Tg*GAD are important enzymes associated with a functional TCA cycle as part of *T. gondii* mitochondrial metabolism. Both are seemingly nonessential, as they can be deleted, and knockout parasites are sufficiently viable for continued culture *in vitro*, but both appear to have a role in conditions where carbon sources are limiting, such as extracellular conditions in between human organs *in vivo*. In this way, *Tg*BCKDH and *Tg*GAD might be critical for extracellular stages to traverse the interorgan spaces to reach the brain and other organs to create cysts.

## Lipid acquisition and synthesis, the importance and flexibility of apicoplast *de novo* synthesis

PEP, and potentially pyruvate generated from glycolysis, can be imported into the apicoplast where, via the FASII pathway, it can be converted to short-chain FAs, primarily C14:0 and C16:0 (Figure 1) [28., 29., 30., 31.]. Ramakrishnan *et al.* showed in *T. gondii* that these short-chain FAs could be transported from the apicoplast to the ER where they can be elongated and desaturated to generate long-chain unsaturated FAs (LCFAs) and very-long-chain unsaturated FAs (VLCFAs) by a series of proteins known as elongases (ELOs) (Figure 1) [28,32]. Three separate ELO homologs (*Tg*ELO-A/B/C) were found to be capable of initiating the conjugation of malonyl-CoA (generated from cytosolic acetyl-CoA) and available FAs [28]. A ketoacyl-CoA reductase (*Tg*KCR) then reduces the substrate, to subsequently be dehydrated by a hydroxyacyl-CoA dehydratase (*Tg*DEH), and reduced once more by an enoyl-CoA reductase (*Tg*ECR) whereby this cycle repeats [32]. The source of carbon required for FA elongation via parasite ELOs is acetyl-CoA. Ultimately provided by cytosolic *Tg*ACS and/or *Tg*ACL, these acetyl-CoA pools are generated using different starting material (Figure 1) (acetate used by ACS, or citrate used by ACL) [17,18]. Both *Tg*ACS and *Tg*ACL are seemingly dispensable individually, but are together essential in *T. gondii* (Table 1) [16], whilst *Pf*ACS is seemingly essential in *P. falciparum* due to the absence of an ACL homolog [18,33].

Individual knockdown of ELOs in *T. gondii* in nutrient-replete conditions had minimal effect on parasite growth [28] whereas knockdown of *Tg*ECR or *Tg*DEH caused severe growth defects (Table 1) [32]. Differentiating the two mutants, *Tg*DEH knockdown growth was partially restored through media supplementation of a mix of various unsaturated VLCFAs and or LCFAs (Table 1) including: C18:1, C20:1, C22:1, and C24:1 [32]. No single unsaturated LCFA or VLCFA would have this same effect, nor a mixture of saturated VLCFAs and or LCFAs [32]. Evidence that the *Tg*ELOs can be depleted individually when parasites are grown in nutrient-replete conditions demonstrates metabolic flexibility in *T. gondii* and warrants further interrogation (Box 1). Genetic knockout of these FA elongation components in *T. gondii in vivo* experimentation has yet to be investigated. Deletion of *PbDEH* in *Plasmodium berghei* has also revealed differing importance to parasite fitness depending on life cycle stage [34,35]. Whether rescue of stages dependent on *PbDEH* is possible through exogenous substrate supplementation *in vitro* has not been thoroughly explored [36]. In addition, there is evidence to suggest that high-intensity mosquito-vector infections can cause parasite intraspecific competition and oocyst dormancy [37]. Under low-intensity infections and with supplementary vector bloodmeals, perhaps local nutrients would be sufficiently rich to ensure DEH-depleted *P. berghei* oocyst survival in spite of its substantial fitness defect.

Box 1 **Interrogating T. gondii FA elongation**

Current evidence suggests that *Tg*ELO-A begins the process of extension from the short FA produced via the apicoplast FASII, with saturated FAs C14:0, C16:0, and C16:1 being converted to C18:0 [28]. Following this first cycle, *Tg*ELO-B then initiates extension of newly formed C18:1 and C20:1 into C22:1, with *Tg*ELO-C beginning the final cycle of extension, converting C22:1 into C26:1 [17,28]. Interestingly, *Tg*ELO-C differentiates itself from *Tg*ELO-A and *Tg*ELO-B by contributing mostly to scavenged saturated FA extension (see Figure 1 in the main text) rather than FASII *de novo*-synthesized [32]. Conditional knockdown of individual *T. gondii* elongases *Tg*ELO-A, *Tg*ELO-B, and *Tg*ELO-C were generated by Ramakrishnan and colleagues, in which no major change in growth rate was identified *in vitro* (see Table 1 in main text) [28]. During this study, attempts to generate a conditional double knockdown of *Tg*ELO-B and *Tg*ELO-C was reported as unsuccessful [28]. Circumventing the challenge, Ramakrishnan later successfully inhibited *Tg*ECR and *Tg*DEH, either of which was sufficient to disrupt the elongation pathway and revealed the fitness contribution of parasite FA elongation to *T. gondii* [32].

Alt-text: Box 1

Beyond FA elongation/desaturation, the fate of apicoplast FAs and their function for the parasite were unknown for a time, and this in regard to the parasite requirement for scavenged FA/lipids [29,32]. Using stable isotope labelling combined with lipidomics approaches, Amiar *et al.* revealed that apicoplast FAs are used to generate the bulk of parasite membrane phospholipids during tachyzoite intracellular development [29]. The study determined that apicoplast FAs accounted for ~60% of the total composition from the major phospholipid classes present in parasite membranes, that is, phosphatidylcholine (PC), phosphatidylethanolamine (PE), and phosphatidylinositol (PI). Furthermore, this revealed that a large majority of phospholipids in tachyzoites are ‘patchwork molecules’ made of one FA *de novo* synthesized by the FASII and the other FA directly scavenged from the host [29]. Therefore, this shows that, in these conditions, both pathways (*de novo* synthesis and scavenging) together critically contribute to parasite membrane biogenesis and intracellular survival [29]. This also indicates that the availability of FA and the proper channeling of these FA based on physiological fluctuations in the host, are central for parasite adaptation to the host lipid availability.

To assemble FAs from both scavenged and *de novo* sources into membrane lipids, *T. gondii* expresses two sets of acyltransferases putatively capable of synthesizing phosphatidic acid (PA), the central precursor for phospholipid *de novo* synthesis by sequential esterification of FAs onto a glycerol 3-phosphate backbone [11]. One set of these acyltransferases is of algal origin and located in the apicoplast *(Tg*ATS1 and *Tg*ATS2), and the second set is eukaryotic and found in the ER (*Tg*GPAT and *Tg*AGPAT) [6,29]. The first enzyme of the pathway, apicoplast glycerol 3-phosphate acyltransferase (*Tg*ATS1), incorporates FAs generated via the FASII to form a lysophosphatidic acid (LPA) precursor (Figure 1) [11]. This LPA precursor is then exported to the ER for further FA elongation and further assembly with scavenged FAs from the host to form the phosphatidic acid (PA) precursor by *Tg*AGPAT that is used for parasite bulk phospholipid synthesis (Figure 1) [11]. Alternatively, LPA made by *Tg*ATS1 can be turned into PA by *Tg*ATS2, but *Tg*ATS2 is dispensable and makes a specific type of short-chain PA rather required for the recruitment of DrpC for cytokinesis [6]. In theory, the ER-localized GPAT can also provide LPA to AGPAT in the ER, but it is not clear why *T. gondii* requires a second ER-localized LPA-synthesizing enzyme, and the full functionality of the ER-localized acyltransferases remains unexplored. Hence, together with the function of the FASII, this shows the importance of the apicoplast to provide essential lipid precursors for parasite survival. The activity of the apicoplast *Tg*ATS1 is also linked to the nutritional content of the host [38]. In limiting FBS conditions, *Tg*ATS1-depleted tachyzoites present a severe defect ablating growth [6,29]. However, with higher host nutritional/lipid content using 10% FBS, lipids scavenged from the host partially compensate for the loss of apicoplast lipids (Table 1) [6].

Based on studies in *T. gondii*, it seems that the apicoplast FASII is needed only to enhance lipid production during growth in low-lipid environments [6]. Consistent with this, it was shown that the metabolic activity of the FASII pathway can be upregulated under host lipid-starved conditions in both *P. falciparum* [39] and *T. gondii* [6]. FabI is the enzyme responsible for the last step of the apicoplast resident FASII, and when a *Pf*FabI KO cell line [40,41] was grown under lipid-starved conditions in blood-stage culture, the mutant died and could not be recovered [6]. Thus, FabI is an example of a FASII component previously believed to be nonessential [40,41], and only when the *Pf*FabI KO was subjected to limiting nutrient conditions outside of traditionally nutrient replete *in vitro* conditions was the importance of *Pf*FabI revealed [6]. Furthermore, these were recently confirmed by other studies, showing that the disruption of the FASII pathway in *T. gondii* resulted in increased dependence on scavenging of external lipid sources in the host environment [42,43].

Historically, the serum content used for *P. falciparum* blood-stage culture media has been varied, with most recent studies using between 1 [44] and 10% Albumax® [6], but the original *P. falciparum* culture studies [45,46] used human serum to grow parasites which has been superseded by Albumax for practical reasons [47]. Similarly, in many *T. gondii in vitro* studies serum concentration in cultures also varies (e.g., 10% FBS [43], 1% or 5% FBS [28,42]). These studies highlight the effect of serum concentration on the experimental outcome; thus, in light of the ability of serum to partially complement a number of metabolic gene KO mutants, the serum concentration added to parasite cultures should be considered in each study in both *Plasmodium* and *T. gondii*. It would be interesting to revisit published deleterious mutants, such as the ELO, DEH, and KCR investigations, to see if these *T. gondii* mutants are essential in 1% FBS or can be compensated under 10% FBS conditions [28]. Another recent study also showed that the extracellular conditions used to grow tachyzoites drives metabolic adaptation, notably by acting upon the apicoplast FASII [48]. Consistent with this, clinical data have shown that parasite apicoplast and FA metabolism genes are upregulated in malnourished patients [49]. This places the parasites back into physiological conditions where the parasite might need the FASII to survive in the blood of a malnourished patient.

We can further extend this concept to consider the variable effect of blood glucose on FASII essentiality, such as in the case of diabetic or obese patients. We would predict that a hyperglycemic environment provides more glucose, so *Plasmodium* (and possibly *T. gondii*) infection would rely more on FASII than scavenging during hyperglycemia, similar to *in vitro* evidence. However, during hypoglycemia, where glucose is low or absent, parasites might rely more on scavenging rather than FASII. It would be interesting to analyze omics data of *Plasmodium* parasites during instances of patient hyperglycemia/hypoglycemia to examine the effect on apicoplast protein expression. Similarly, comorbidity with HIV-AIDS is a common problem during malaria [50] and toxoplasmosis [51]. An absent immune response would likely make exported effectors less important for parasite growth due to the lack of a robust immune response and may require more efficient drugs to kill parasites due to these favorable host environmental conditions [51,52]. It would be interesting to analyze both *Plasmodium* and *T. gondii* transcriptomic data during coinfection with HIV-AIDS to examine the effects of immunosuppressed host environments on parasite metabolic gene expression and expression of exported host effectors.

## Scavenging from the host environment and inducing differentiation in apicomplexan parasites

The FASII proteins of the relict plastid are conserved in well studied plant models and are easily identifiable; however, exported host effectors are relatively poorly conserved, and progress has been slower to identify scavenging components in both *T. gondii* and *Plasmodium* [53]. Early research on *T. gondii* host effectors found the highly immunogenic dense granule effectors GRA1–GRA10 important for the maintenance of the parasitophorous vacuole within the host cell [54,55]. A later study on parasite Golgi-resident aspartyl protease (*Tg*ASPV) found that this protein was important for the trafficking of various exported host effectors in *T. gondii* [56., 57., 58.]. However, effects of deletion of these genes on lipid metabolism during scavenging have been largely overlooked. Recently the *Tg*ASPV KO mutant was shown to grow less efficiently under low-lipid host environments (Table 1) [6], which suggests that virulence of Δ*Tg*ASPV tachyzoites might be further reduced in mice fed a calorie-restricted diet. This could have important consequences for drug development of the *Plasmodium* homologue plasmepsins during blood-stage growth [59].

Scavenging from the host is an important adaptation response to FASII inhibition [6,28]. Therefore, it appears that the parasite can also regulate its scavenging ability; however, detailed mechanisms of this regulation are unknown, and even the discovery of upregulation of scavenging is recent. It was shown that *T. gondii* tachyzoites can induce the formation of **giant multivesicular bodies** (**gMVBs**) in **human foreskin fibroblasts** (**HFFs**) grown in limited serum – a parasite response to aggressively upregulate lipid scavenging of host organelles including the ER and even the host nucleus [6]. Similarly, it has been known for many years that the apicoplast is responsible for synthesizing short-chain FAs C14:0 and C16:0 (Figure 1) [28], but a lipid scavengeome has only recently been identified [38]. By growing *T. gondii* parasites on ^13^C-labelled host cells, it was discovered that the majority of key FAs scavenged were 16:0, 16:1, 18:0, and 18:1 [38]. Additionally, only a minor amount of C14:0 was scavenged [38], consistent with reports that scavenging only partially compensates for the apicoplast FASII disruption [6,28]. This demonstrates that apicoplast FASII and scavenging are together responsible for specific FA classes which complement each other to form a hybrid mix of lipids, consistent with published reports on phospholipid production in *T. gondii*.

To channel the constant flux of scavenged host lipids for membrane biogenesis, and to avoid death by lipotoxicity (i.e., excess of lipids), the parasite has a single lipin (i.e., *Tg*LIPIN, a PA phosphatase) at the cytosol–ER interface (Figure 1) [38]. *Tg*LIPIN is essential for lipid homeostasis, maintaining a metabolic balance (membrane biogenesis versus storage) to regulate constant flux of scavenged lipids and parasite survival by allowing the incorporation of host FAs into storage lipids, triacylglycerol, which are then mobilized specifically during parasite division (Figure 1) [38]. *Tg*LIPIN is also largely influenced by the host nutritional environment [38]. Conversely to *Tg*ATS1, the absence of *Tg*LIPIN induces a much stronger growth defect at high-host-lipid content (10% FBS) than at low-lipid content (Table 1) [1% FBS in Dulbecco's Modified Eagle Medium (DMEM)] [38], showing that certain genes are required to adapt to a high-lipid environment and others (like *Tg*AST1) to adapt to a low-lipid environment [38]. This would also have important consequences for human infection where perhaps *Tg*ATS1 might be not essential in a lipid-rich environment such as an adipocyte [60] but rather highly essential in a liver cell, which is a major site of gluconeogenesis in the human body where glucose would be the primary carbon source [61]. Conversely, *Tg*LIPIN would be predicted to be essential in an adipocyte where excess free FAs could cause lipotoxicity if not stored properly in tachyzoites.

Similar research on serum availability in *T. gondii* type II strains ME49 and PRU, that more readily differentiate into bradyzoites, showed that titrating down FBS in the medium results in reduced growth [6]. Potentially, this may be due to the formation of slow-growing, dormant bradyzoites caused by low nutrient availability, in this case exogenous lipids [6]. In this way, it appears that generally starving the parasite of a major nutrient stresses the tachyzoites and induces stage conversion into the bradyzoite stage, although further research is needed to validate the effect of lipid starvation for stage conversion [6,8,9].

Similarly, *Plasmodium* sexual conversion is sensitive to levels of lysophosphatidylcholine (LPC) [62], an important lipid component of human serum and source of building blocks for parasite membrane biogenesis during asexual division. Changes in LPC levels represent a major change in the environmental nutrient composition and can be seen as a sort of ‘environmental cue’ triggering stage conversion by limiting access to an important nutrient for propagation [62], somewhat similarly to *Toxoplasma* bradyzoites. A similar conversion has recently been established for *T. gondii* sexual-stage conversion where its sexual-stage conversion is dependent on the levels of linoleic acid (18:2) in the feline host environment [63]. Linoleic acid levels are high in feline intestinal tracts due to the absence of a FA desaturase, thus serving as an environmental cue for sexual-stage conversion in the definitive feline host [63]. In this way, the host nutrient environment can be seen as a very important determinant of stage conversion allowing the parasite to ‘decide’ to convert or not consistent with a role in determining which parasite genes are essential for survival.

## Scavenged amino acids, and the role of amino acid transporters

*T. gondii* is auxotrophic for most amino acids and needs to scavenge them from the host cell to survive [18]. Hence, the amino acid availability of the host environment can be seen as another key determinant of the viability of *T. gondii* parasites. For example arginine has been shown to be necessary for tachyzoite growth [8], and conversely, the loss of this amino acid has been shown to induce differentiation of otherwise rapidly replicating *T. gondii* tachyzoites to form slow-growing bradyzoites [64]. It is conceivable and likely that bradyzoite conversion would also occur under deprivation of tryptophan or certain vitamins [42]. Surprisingly however, this does not appear to be the case for *T. gondii’*s largest source of carbon, glucose [65]. Further work is needed to address if there are any major nutrients that can be deprived without triggering bradyzoite formation.

In the acute infection stage, amino acid scavenging is important for rapid tachyzoite growth evidenced by very recent discoveries on the mechanisms of amino acid scavenging [8,9,66., 67., 68., 69.]. The first such transporter identified was an amino acid transporter *Tg*NPT1 (later renamed as apicomplexan amino acid transporter *Tg*ApiAT1) which primarily transports arginine from the host (Figure 1) [8]. **Roswell Park Memorial Institute (RPMI) medium** growth medium typically contains different amino acid composition than DMEM growth medium, and the *Tg*APiAT1-depleted cells grow normally in RPMI (Table 1) [8]. However, when Δ*Tg*ApiAT1 mutants were grown in DMEM medium, Δ*Tg*ApiAT1 tachyzoites grew poorly. The substrate affinity of the *Tg*ApiAT1 protein was investigated through ^14^C isotopic labelling experiments on *Xenopus* oocytes and found to primarily transport arginine, and so differences in arginine content between RPMI and DMEM was responsible for this difference in Δ*Tg*ApiAT1 growth [8]. These data indicate that amino acid concentration (in this case arginine) of the environmental medium determines the essentiality of the *Tg*APiAT1 gene [8].

Other amino acid transporters have since been discovered, including *Tg*ApiAT5-3, a tyrosine transporter (Figure 1) [66]. Addition of exogenous excess tyrosine improved growth of *Tg*ApiAT5-3 KO mutants (Table 1), which suggests the presence of a complementary transporter also capable of importing tyrosine, similar to the observations of *Tg*APiAT1 [66,68]. *Tg*ApiAT5-3 was also shown to have some affinity for aromatic amino acids phenylalanine and tryptophan [66,68], although there is likely another complementary transport pathway whose primary role is to import phenylalanine and tryptophan (Figure 1) [68]. Recently *Tg*ApiAT6-1 was found to primarily transport lysine [9,69] but was also found to have a minor affinity for arginine (Figure 1) [9]. In isotopic labelling studies *Tg*ApiAT6-1 was demonstrated to be responsible for importing arginine during the loss of *Tg*ApiAT1, and so co-operates depending on the availability of both arginine and lysine in the host environment [9]. This explains how *Tg*ApiAT6-1 can compensate for the loss of *Tg*ApiAT1 when *T. gondii* is grown with excess arginine in the medium. Likely a similar mechanism exists for *Tg*ApiAT5-3 and aromatic amino acids [68]. Crucially, the expression of *Tg*APiAT1 and the abundance of the *Tg*APiAT1 protein has been shown to increase with the scarcity of arginine in the environment, meeting tachyzoite growth requirements [67]. This is seemingly the first known example of a *T. gondii* transporter whose regulation is directly dependent on its substrate availability. In this way, the expression of *Tg*ApiAT1 is higher or lower in host cell types that have higher or lower levels of arginine availability. For example, liver cells are low in arginine due to the expression of an arginine-degrading enzyme, arginase [70]. In a subsequent mouse-infection experiment, expression of *Tg*ApiAT1 was found to be lower in kidney and spleen cells whereas *Tg*ApiAT1 expression was relatively higher in mouse lung tissue, the peritoneal cavity and, predictably, liver cells where arginine abundance is relatively lower [67,70,71]. This sets a precedent whereby parasite gene essentiality/importance could potentially be predicted depending on the nutrient content of the host cell environment validating *in vitro* and *in silico* [6] experiments *in vivo* [67]. Therefore, based on these data, we would predict that *Tg*ApiAT1 would be relatively more important for parasite growth during infection of liver cells.

## Concluding remarks

In summary, parasites are adaptable to host (nutritional) environments, and it is important to consider gene or pathway essentiality in this context. A good example was recently provided in human cells, in which basal and serum components of the culture media influenced gene essentiality scores in a **clustered regularly interspaced short palindromic repeats (CRISPR)** screen [72]. This is likely reflected also in the differences between essentiality scores of *T. gondii* genes in a CRISPR screen performed in culture under nutrient-replete conditions [73] or in animals [74,75] or specific drug selection [76]. Such metabolic studies in *T. gondii* and in *Plasmodium* sp. would allow us to predict gene essentiality of parasites based on the host nutrient content. This may have consequences for prioritization of targets for drug discovery. In the example we provided about the roles of the apicoplast FASII pathway, it is relevant to consider that a patient suffering from malnutrition is more likely to have low lipid availability in their blood for the parasites to scavenge [77]. This may highlight the apicoplast FASII as a relevant drug target for malaria [78], despite the findings pointing to IPP synthesis as the only essential apicoplast function in nutrient-rich *in vitro* blood-stage studies [79]. Immunologists have known for a long time about the limitations of *in vitro* culture studies due to the absence of a robust host immune response [74,80,81]; however, external/growth environments have often been simply viewed as a food source. The recent literature, however, shows that the metabolic relationship between parasite and host is in fact equally complex (see Outstanding questions). Accordingly, in *vivo* screens in rodent malaria have yet to fully explore the effects of caloric restriction on infected host mice, which could bias the data [7,36]. As large-scale studies become cheaper and more accessible, we predict that different growth conditions will serve as a great way to interrogate the entire genome for the variable essentiality of metabolic genes under specific growth conditions, and possibly identify previous overlooked drug targets that might work in combination therapy to complement clinical studies to treat these diseases.

Outstanding questions How can we adapt genome-wide *in vitro* KO screens to limit bias from environmental nutrient conditions and avoid overlooking potential drug targets?

Which cell culturing conditions and media compositions are representative of specific host tissue environments that apicomplexan parasites infect *in vivo*?

Potential metabolic drug targets may have been abandoned for not showing parasite fitness defects *in vitro* under high-nutrient conditions*.* Which of these targets are worth revisiting?

Could the apicoplast FASII still be a relevant drug target for *Plasmodium?*

Can apicomplexan parasite infection be modulated by caloric restriction in mouse studies?

Alt-text: Outstanding questions

## Glossary

**Clustered regularly interspaced short palindromic repeats (CRISPR)**: a genetic engineering tool permitting targeting of specific sequences for genome editing. Different CRISPR-associated systems or Cas proteins can be used to introduce single- or double-stranded breaks in DNA or RNA.
**ΔPYK2**: a *T. gondii* cell line permanently deficient in the pyruvate kinase 2 gene. Delta is regularly used to symbolize a genetic deletional mutant of the gene that follows the symbol.
**Dulbecco's Modified Eagle Medium (DMEM)**: a medium commonly used for culturing mammalian cells.
**Fetal bovine serum (FBS)**: serum derived from a bovine fetus. Commonly used in cell cultures as a source of lipids, growth factors, and other nutrients.
**Giant multivesicular bodies (gMVBs)**: large membranous structures enclosing multiple individual compartmentalized vesicles. These structures were first described in host cells infected with *T. gondii* and cultured in low serum content media.
**Human foreskin fibroblasts (HFFs)**: a durable cell line, derived from human foreskin, commonly used as a host cell monolayer for culturing the parasite *T. gondii*.
**iΔPYK1**: a promoter replacement insertional *T. gondii* mutant targeting the parasite’s pyruvate kinase 1 gene. Under experimental conditions, exogenous supplementation of tetracycline was reported to temporarily repress PYK1 gene expression and thus activity.
**Knockdown**: to reduce expression of a gene of interest in an organism under experimental conditions. A term commonly associated with RNA interference gene silencing techniques but can also refer to promoter-insertion methodology.
**Knockout (KO)**: a genetically modified organism that has had a gene of interest deleted.
**(Liverpool) Library of Apicomplexan Metabolic Pathways (LAMP)**: an online database mapping metabolic pathways of several Apicomplexan parasites, including *T. gondii*.
**Phosphate-buffered saline (PBS)**: a common laboratory buffer. It is osmotically balanced to reduce the risk of cell desiccation or lysis but is otherwise nutritionally deficient.
**Roswell Park Memorial Institute (RPMI) medium**: a medium commonly used for culturing mammalian cells.
